# Complete SLC4A11 Detection in Saudi Congenital Hereditary Endothelial Dystrophy: A Transmembrane Glycine Hotspot and a Recurrent Splice Donor Allele in Consanguineous Patients

**DOI:** 10.3390/ijms27167220

**Published:** 2026-08-13

**Authors:** Khaled K. Abu-Amero, Rizwan Malik, Sara M. AlHilali, Deema E. Jomar

**Affiliations:** 1 Research Department, King Khaled Eye Specialist Hospital and Research Center, Riyadh 11462, Saudi Arabia; 2 Anterior Segment Division, King Khaled Eye Specialist Hospital and Research Center, Riyadh 11462, Saudi Arabia

**Keywords:** SLC4A11, congenital hereditary endothelial dystrophy, corneal dystrophy, consanguinity, whole-exome sequencing, Saudi

## Abstract

Congenital hereditary endothelial dystrophy (CHED) is a rare autosomal recessive disorder of the corneal endothelium caused by biallelic variants in the *SLC4A11* gene. Its genetic spectrum is well described in the Indian subcontinent, but poorly characterized in the Saudi population, where high rates of consanguinity may concentrate specific alleles. We performed whole-exome sequencing in 47 clinically diagnosed CHED patients from consanguineous Saudi families and classified the resulting variants by their predicted effect on the protein. A homozygous *SLC4A11* variant was identified in every patient, and no compound heterozygotes were found. The 47 patients carried 20 unique variants, three of which were novel: c.1799C>A; p.A600E, c.2623C>T; p.R875*, and c.670dup; p.W224Lfs*7. Seven variants recurred, and the canonical splice donor change c.2018+1G>A alone accounted for 14 patients. The variants resolved into two mechanistic groups: 22 patients with missense substitutions predicted to misfold the protein and cause endoplasmic reticulum retention, and 25 with truncating or splice variants predicted to abolish the protein. Among those, only the p.R875* sequence variant removes only the final 17 residues, is predicted to escape nonsense-mediated decay, and likely leaves a near-full-length protein. Five of the nine missense variants were glycine-to-arginine substitutions clustered between residues 378 and 413, within the first transmembrane region. The complete detection rate supports genetic homogeneity of CHED in this population and contrasts with the substantial fraction of unsolved cases reported elsewhere. The concentration of variants in a few regions of the gene suggests that a targeted Sanger protocol covering these hotspots could serve as an affordable first-line diagnostic test, with exome sequencing reserved for hotspot-negative patients.

## 1. Introduction

CHED has been historically divided into two types. Type 1 is an autosomal dominant disorder that manifests after the first year of life and is now classified as a variant of posterior polymorphous corneal dystrophy (PPCD) [1]. Type 2 is an autosomal recessive disorder that manifests at birth and is caused by mutations in the *SLC4A11* gene. Currently, ophthalmologists typically use the name CHED exclusively to denote the autosomal recessive variant (formerly referred to as CHED 2). The epidemiology of CHED differs by geographic region and seems to be affected by consanguinity patterns. Numerous genetic investigations on CHED have been performed in populations from the Indian subcontinent, where multiple families with solute carrier family-4-member 11 (*SLC4A11*) gene mutations have been documented, and a broad array of pathogenic variations has been characterized [2,3,4]. Conversely, there is a paucity of genetic evidence from Middle Eastern populations. In Saudi Arabia, where consanguineous marriages are prevalent, autosomal recessive syndromes like CHED may manifest with increased frequency. A high number of *SLC4A11* mutations, including a number of novel variants, have been reported in previous studies of Saudi patients with CHED [5], suggesting the existence of population-specific mutation patterns. Histopathologically, the disease is characterized by degeneration or absence of corneal endothelial cells, stromal edema, and Descemet’s membrane thickening [6]. The corneal endothelium plays an important role in maintaining corneal clarity by regulating stroma hydration via barrier and pump functions that mediate fluid transport from corneal stroma to aqueous humor. Malfunction of these systems results in excessive stromal hydration, disarray of collagen fibrils, and ensuing corneal opacity. Despite the promising nature of ongoing research into innovative genetic and mechanism-based nonsurgical therapies, corneal transplantation, encompassing penetrating keratoplasty or endothelial keratoplasty, continues to be the sole effective treatment for restoring corneal clarity and visual function in affected individuals [7]. Mutations in the *SLC4A11* gene are recognized as the principal cause of autosomal recessive CHED [8,9]. The *SLC4A11* gene codes for bicarbonate transporter-related protein 1 (BTR1), which belongs to the SLC4 family of membrane transport proteins. This protein possesses multiple transmembrane domains and is expressed in several tissues, such as the corneal endothelium, where it plays an essential part in sustaining ionic homeostasis and fluid transport across the endothelial layer. Alterations in SLC4A11 lead to impaired endothelial cell function and ionic transport pathways, resulting in aberrant regulation of corneal hydration and stromal edema, a hallmark of CHED [10]. In addition to CHED, mutations in *SLC4A11* have been linked to various ocular and systemic disorders, including homozygous mutations causing corneal dystrophy with perceptive deafness (Harboyan syndrome) and heterozygous mutations observed in some instances of late-onset Fuchs endothelial corneal dystrophy. Experimental studies have suggested that *SLC4A11* may function as an ion transporter or channel that mediates the transport of sodium, protons, and water across cell membranes, underlining its significance in corneal endothelium physiology and fluid homeostasis. Subsequent to the identification of the role of *SLC4A11* in the pathogenesis of CHED, several pathogenic variants have been reported worldwide. The mutations include missense, nonsense, splice-site, and insertion-deletion variants distributed throughout the gene, many of which are predicted to affect protein folding, intracellular trafficking, or membrane localization. Although *SLC4A11* is the only gene conclusively linked to CHED, the inability to detect pathogenic SLC4A11 mutations in some patients points to the possible involvement of other deleterious genetic mutations. A homozygous mutation in the *MPDZ* gene, which encodes a protein with multiple PDZ domains, was identified in a patient with clinically proven CHED who did not carry an *SLC4A11* mutation [11]. A homozygous *FAM149A* mutation (c.991A>G; p.R331G) was suggested as a potential pathogenic factor in a patient with CHED who carried non-pathogenic *SLC4A11* polymorphisms. These studies suggest that the genetic basis of CHED is poorly understood, and more research using methodologies such as whole-exome sequencing is needed to examine other possible mutant pathogenic variations. Identification of additional causal genes may improve understanding of the molecular pathways associated with corneal endothelial dysfunction. The clinical differentiation of CHED from other causes of corneal opacification in infancy is challenging, and its association with primary congenital glaucoma (PCG) has been well documented. Both conditions can present with bilateral corneal edema, opaque corneas, and elevated intraocular pressure readings, the latter of which are erroneously increased due to the increased central corneal thickness seen in CHED. Several reports have described patients with *SLC4A11* mutations who were initially misdiagnosed with primary congenital glaucoma (PCG) for years, undergoing multiple unnecessary glaucoma surgeries, until genetic testing corrected the diagnosis to congenital hereditary endothelial dystrophy (CHED) [12,13]. This overlap emphasizes the need for genetic confirmation for accurate diagnosis and management. In a preliminary screening of consanguineous CHED families, *SLC4A11* mutations were identified in all affected individuals in the Saudi population, all in the homozygous state and all predicted to reduce or abolish BTR1 function, with no evidence of a second causative locus [5]. Whether this apparent genetic homogeneity exists in a larger and more diverse Saudi cohort remains to be determined. The genetic spectrum of CHED in the Saudi population remains undefined. We therefore screened patients with a clinically confirmed diagnosis of CHED for pathogenic variants in *SLC4A11*, aiming to identify recurrent alleles that could form the basis of a rapid, targeted diagnostic pipeline.

## 2. Results

### 2.1. Clinical Findings

**Figure 1:**
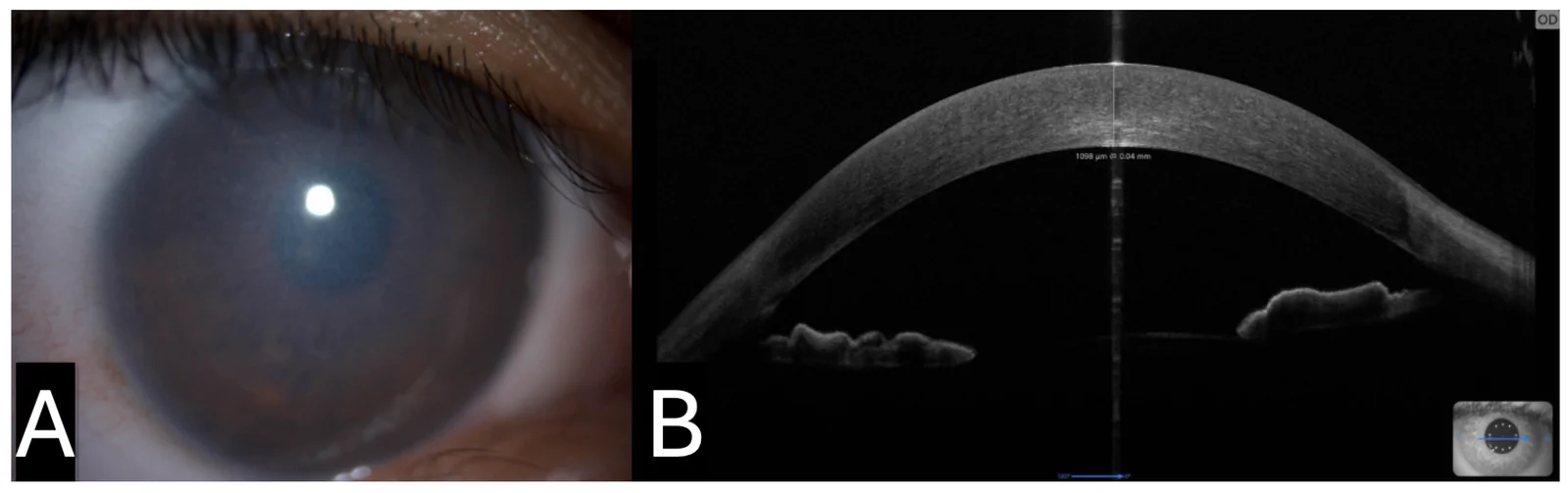
(**A**) Slit lamp image of the right eye of one patient from the study cohort showing diffuse limbus to limbus corneal haze with obscured iris details; (**B**) Anterior Segment Optical Coherence Tomography (AS-OCT) showing diffuse corneal thickening with increased stromal hyper-reflectivity consistent with CHED.

There were 47 Saudi patients in all, 25 of whom were female and 22 of whom were male. Their current ages ranged from 4 to 59. Examining the medical records, the majority of patients started developing symptoms at an average age of less than a year, which corresponds to the age at which congenital CHED symptoms usually appear. Slit-lamp examination demonstrated diffuse ground-glass corneal haze involving the entire cornea with a mosaic appearance, markedly increased central corneal thickness, and the absence of buphthalmos, consistent with CHED. Parental consanguinity was common, with 40 (85.1%) patients born to first-degree consanguineous parents, 5 (10.6%) to second-degree consanguineous parents, and 2 (4.3%) to unrelated parents (as per patient report). A positive family history of CHED was present in 34 (72.3%) patients, while 13 (27.7%) had no known family history. Ten patients had documented hearing loss. Reviewing patients’ history, age at presentation ranged from 1 month to 17 years. All patients showed the classic CHED phenotype on examination, with bilateral diffuse corneal edema present from infancy. A representative example, with anterior segment ocular coherence tomography findings, is shown in Figure 1.

**Figure 2:**
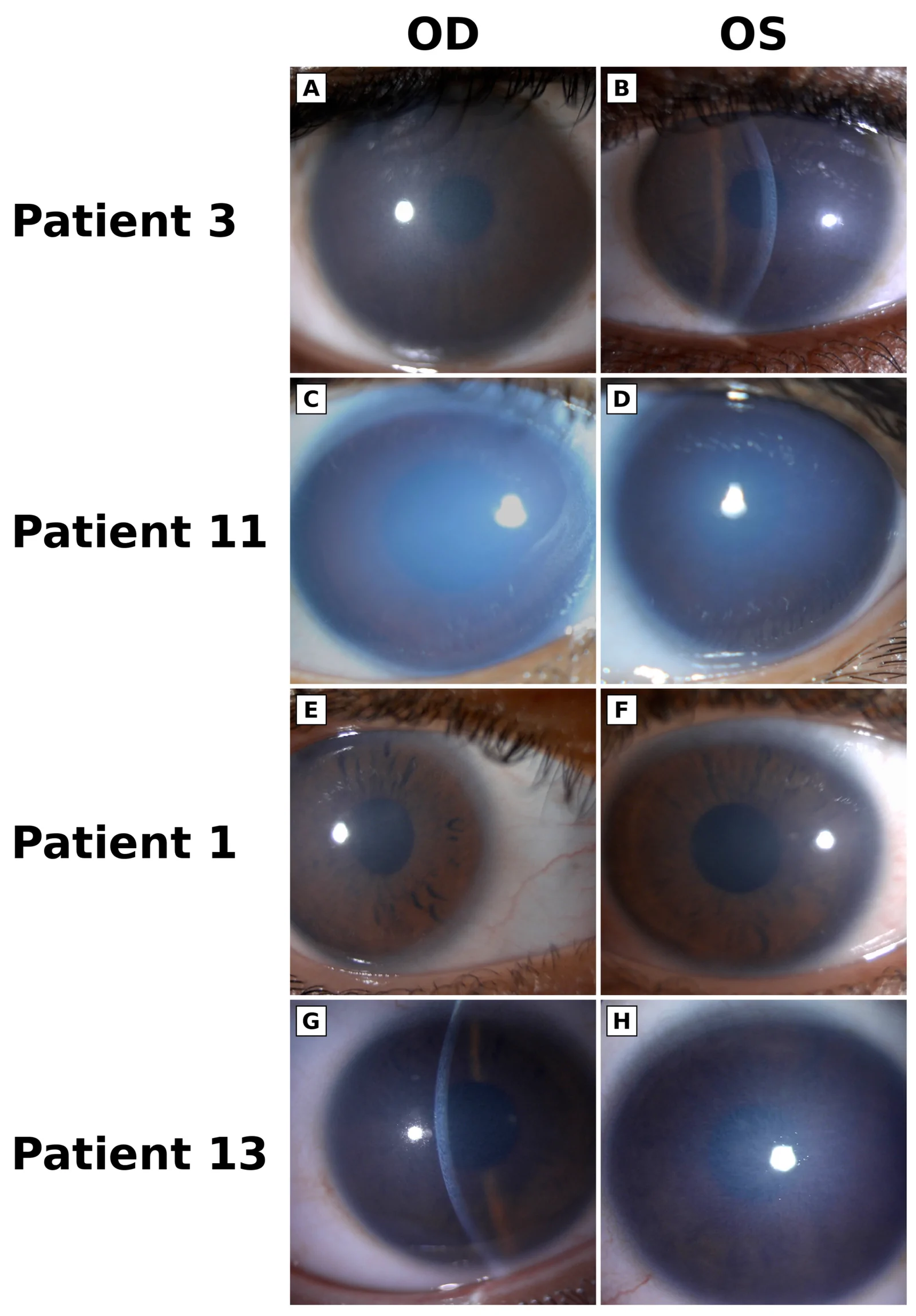
Clinical anterior segment photographs of four typical patients in our series to demonstrate the phenotypic variability in our study. Slit lamp photographs of the right, **OD** (**A**,**C**,**E**,**G**) and left, **OS** eyes (**B**,**D**,**F**,**H**) are shown. **Patient 3** (CHED-3) is 17-year-old girl (diagnosed with CHED at the age of five years old) with moderate corneal haze OU, corneal thicknesses of around 650 µmin each eye with BCVA of 20/80 OD and 20/40 OS; **Patient 11** (CHED-11) is 13-year-old boy (diagnosed aged 2 years) showing appearances aged 4 years old, with severe corneal haze in both eyes prior to posterior lamellar grafts (DSAEK) in both eyes; **Patient 1** (CHED-1) is a 6-year-old boy (diagnosed with CHED aged 2 months old), with mild corneal haze OU, corneal thickness of over 1000 µmin each eye and BCVA of 20/200 OD and 20/80 OS; **Patient 13** (CHED-13) is an 11-year-old boy (diagnosed aged 2 months) with mild corneal haze OD and moderate corneal haze OS, corneal thickness of over 1000 µm in each eye and 20/100 OD and 20/80 OS. BCVA = best corrected visual acuity; CHED = corneal hereditary endothelial dystrophy; DSAEK = Descemet’s Stripping Automated Endothelial Keratoplasty. OU = both eyes.

Phenotypic variability of the cornea for four typical patients from the cohort is shown in Figure 2. The four patients illustrate the phenotypic range seen across our CHED cohort, from mild to severe corneal haze at presentation. Severity of haze does not track cleanly with corneal thickness or visual acuity. CHED-1, graded as mild haze, had the thickest corneas (over 1000 µm) and the worst acuity (20/200 OD), while CHED-3, graded as moderate, had thinner corneas (650 µm) and better vision. The figure shows that clinical severity in CHED is not captured by any single measure (Figure 2).

### 2.2. Genetics Findings

We screened 47 clinically confirmed CHED patients (each patient represents one unique family) and detected a homozygous *SLC4A11* variant in everyone. No compound heterozygotes were found. The 47 patients carried 20 unique *SLC4A11* variants. Seven of these recurred. The canonical splice donor change c.2018+1G>A was by far the most common and accounted for 14 patients, close to a third of the cohort. (c.1132G>C; p.G378R) and (c.1180G>C; p.G394R) were each detected in 5 patients, (c.700G>T p.V234F) in 4 patients, and (c.1189G>A; p.G397R), (c.2140C>T; p.R714*) and (c.764C>T; p.T255M) in 2 patients each. The remaining 13 variants were each seen in a single patient. By variant class, 22 patients carried a missense change, 17 carried a splicing change, 6 carried a nonsense change, 1 carried a small deletion, and 1 carried a duplication. Counted as unique variants, the distribution was 9 missense, 5 nonsense, 4 splicing, 1 deletion and 1 duplication. The missense variants were therefore more diverse, while the splicing variants were more recurrent. Mechanistically, the cohort splits in two. Twenty-two patients carry missense substitutions predicted to misfold the protein or disrupt transmembrane domain 1. The other 25 carry variants predicted to truncate the protein or abolish a canonical splice donor site, a group dominated by the 14 patients with c.2018+1G>A and the resulting exon 16 skipping. By pathogenicity, 25 patients carried a likely pathogenic variant, 18 carried a pathogenic variant, and 4 carried a variant of uncertain significance (VUS). Counted as unique variants, 8 were likely pathogenic, 8 were pathogenic, and 4 were of uncertain significance. Three variants appear to be novel. These are c.1799C>A; p.A600E (missense, uncertain significance, CHED-03), c.2623C>T; p.R875* (nonsense, uncertain significance, CHED-53) and c.670dup; p.W224Lfs*7 (duplication, likely pathogenic, CHED-43). The other 17 unique variants have been reported previously (Table 1).

We grouped the 20 unique variants by their predicted effect on the SLC4A11 protein. We detected two mechanistic classes that accounted for the whole cohort. Class 1 comprised the nine missense variants found in 22 patients, all predicted to produce a full-length protein that misfolds and is retained in the endoplasmic reticulum rather than reaching the plasma membrane. Class-2 comprised the 11 truncating and splice variants found in the remaining 25 patients, in which the protein is predicted to be lost through premature termination, nonsense-mediated decay or loss of a canonical splice donor. Within Class 1, five of the nine missense variants were glycine-to-arginine substitutions clustered between residues 378 and 413, spanning the start of the membrane domain and the first transmembrane segment, and these five alone accounted for 14 of the 22 missense patients, while only p.V234F and p.T255M fell in the N-terminal cytoplasmic domain. Within Class-2, the canonical donor variant c.2018+1G>A dominated with 14 patients, and p.R875* stood apart from the other nonsense variants because it removes only the final 17 residues of the 891-residue protein and is predicted to escape nonsense-mediated decay, which is the basis for its classification as a variant of uncertain significance. All effects reported in Table 2 are predictions based on variant position and type and were not confirmed by trafficking, transport or RNA assays in this study (Table 2).

## 3. Discussion

We screened 47 clinically confirmed CHED patients from consanguineous Saudi families and identified a homozygous *SLC4A11* variant in every CHED patient we screened. This 100 percent detection rate is the central result. It stands in contrast to reports from the Indian subcontinent, where pathogenic *SLC4A11* variants were absent in a substantial fraction of CHED families, ranging from 17 to 55 percent across four separate cohorts [8,13], and to the Iranian series in which one of 20 probands carried no *SLC4A11* variant and was instead attributed to *MPDZ* [11]. Our data align with the earlier, smaller Saudi study of Aldahmesh and colleagues, who also identified causative *SLC4A11* variants in all 10 patients across seven families and argued for genetic homogeneity of CHED in this population [5]. The present cohort, roughly five times larger, strengthens that conclusion. In the Saudi population, a negative *SLC4A11* result should prompt review of the clinical diagnosis before a search for a second locus. All 47 patients were homozygous, with no compound heterozygotes. This is the expected signature of disease arising through consanguinity rather than through independently inherited alleles, and it matches the homozygous state reported in the earlier Saudi [5] and the Iranian cohorts [11]. The high proportion of recurrent variants points the same way. Seven of our 20 variants recurred, and the canonical splice donor change c.2018+1G>A alone accounted for 14 of 47 patients, close to a third of the cohort. A small number of alleles reaching this frequency in unrelated families may be consistent with founder effects, a pattern already described for *SLC4A11* in the region, including the p.E675A allele proposed as a founder mutation in the neighboring Pakistani population [14]. Whether c.2018+1G>A represents a Saudi founder allele will require haplotype analysis, which we did not perform. Grouping the variants by their predicted effect on the protein resolved the cohort into two mechanistic classes. Twenty-two patients carried missense substitutions predicted to misfold the protein and cause retention in the endoplasmic reticulum, and 25 carried truncating, frameshift or canonical splice variants predicted to abolish the protein through premature termination or loss of a splice donor. Both routes converge on the same endpoint, absence of functional bicarbonate transporter–related protein 1 at the endothelial plasma membrane, which is the mechanism repeatedly proposed for CHED [3,5]. Notably, five of our nine missense variants were glycine-to-arginine substitutions clustered between residues 378 and 413, at the start of the membrane domain and the first transmembrane segment. This includes p.G394R, the same substitution Aldahmesh and colleagues assigned to disruption of transmembrane domain 1 in a Saudi proband [5], which suggests this short stretch is a recurrent target of pathogenic missense change in this population. The introduction of a bulky, charged arginine in place of a small, flexible glycine within a membrane-embedded helix is a plausible driver of misfolding and quality-control degradation. Three variants appear novel: c.1799C>A; p.A600E, c.2623C>T; p.R875*, and c.670dup; p.W224Lfs*7. We classified the two missense changes in uncertain significance, p.A600E and p.E659K, conservatively, since neither has functional support. The nonsense variant p.R875* also received a VUS call, on the basis that it removes only the final 17 residues of the 891-residue protein and is predicted to escape nonsense-mediated decay, so a near full-length product may persist. This mirrors reasoning applied elsewhere to C-terminal truncations of *SLC4A11*, where residual protein complicates the interpretation of otherwise clearly truncating alleles [15]. We did not perform trafficking, transport or RNA studies, so all effects reported here are predictions from variant position and type, and the mechanistic classes should be read as hypotheses rather than demonstrated outcomes. The clinical data reinforce a point of practical importance: the overlap between CHED and primary congenital glaucoma [16]. Our patients spanned a wide range of recorded ages, and several had undergone earlier intervention. The Pakistani study of Yousaf and colleagues documented a family with CHED2 misdiagnosed and treated as congenital glaucoma for nine years before whole-exome sequencing corrected the diagnosis [12], and elevated intraocular pressure readings in CHED are a recognized source of this error, since a thickened, edematous cornea can produce falsely high tonometry. Genotype alone did not predict clinical course, an observation also made in the Iranian and Indian cohorts, where no consistent genotype–phenotype correlation for disease severity emerged [3,4]. This argues for genetic confirmation in every infant with bilateral corneal clouding, both to separate CHED from glaucoma and to avoid unnecessary glaucoma surgery. All variants reported here were identified by whole-exome sequencing, an approach that is comprehensive but remains costly and slow for routine diagnostic use in a high-volume setting. The distribution of variants in this cohort suggests a practical alternative. Because the Saudi mutational spectrum is dominated by a small number of recurrent alleles concentrated in a few regions of the gene, most patients could be diagnosed with a targeted Sanger protocol covering those regions alone. Four amplicons would suffice: one spanning c.700 to c.764, one spanning the c.1132 to c.1237 cluster in the transmembrane domain 1 region, one covering the c.2018+1 canonical splice donor, and one spanning c.2118 to c.2188. Together, these regions account for 39 of our 47 patients, or 83 percent. Adding a fifth amplicon across the c.1282 donor site raises the yield to 41 of 47, or 87 percent. A tiered strategy therefore becomes feasible in which Sanger sequencing of these hotspots is performed first, at a fraction of the cost and turnaround of exome sequencing, and only hotspot-negative patients proceed to full-gene or exome analysis. The residual 13 percent of our cohort carried private variants scattered across the gene, including the two frameshift alleles, which is why a negative hotspot screen must reflex to broader testing rather than being reported as uninformative. The hotspot definition is population-specific and derived from a single center, so it would require prospective validation in an independent Saudi series before adoption as a first-line diagnostic test. We explored whether the *SLC4A11* mutation class (missense versus nonsense variants) was associated with the severity of the native corneal phenotype. The native pre-treatment phenotype was reconstructed using the earliest available best-corrected visual acuity (BCVA) and Ramappa clinical grading of archived slit-lamp photographs, as most patients had subsequently undergone corneal transplantation, precluding direct assessment of the original disease severity. Interpretation of these findings was limited by several methodological constraints. Complete preoperative phenotypic data were available for only 15 of the 47 patients, with marked class imbalance between mutation groups (3 nonsense and 12 missense variants). In addition, Ramappa grading was performed retrospectively using sill lamp photographs rather than standardized clinical examinations, making the assessment susceptible to differences in image quality, illumination, exposure, and focus. The photographs were also obtained at varying ages, introducing potential confounding from age-related changes in disease expression. Thus, it was not possible to establish any reliable statistical correlation between genotypic classification and clinical phenotype. To properly evaluate genotype-phenotype relationships in this cohort, a prospective study with standardized clinical assessment protocols would be needed. Beyond these limitations, comprehensive genotype-phenotype correlation necessitates deployment of advanced genomic methodologies beyond targeted exome sequencing performed here. Whole-genome sequencing would enable systematic interrogation of the complete genomic landscape to identify potential modifier genes or specific SNP haplotypes that may modulate disease severity. Such regulatory variants and genetic interactions could elucidate the mechanistic basis underlying phenotypic heterogeneity observed among *SLC4A11* mutation carriers. Integration of polygenic risk assessment and haplotype analysis would provide the requisite genetic resolution to establish robust genotype-phenotype associations in this patient population. This study has limitations. We did not carry out functional validation, so the two mechanistic classes rest on prediction. Haplotype analysis, which would confirm or exclude a founder origin for the recurrent alleles, was not performed. In summary, we describe the largest Saudi CHED cohort reported to date. The complete detection of homozygous *SLC4A11* variants supports genetic homogeneity of the disease in this population, identifies a recurrent glycine-to-arginine missense cluster and a common splice donor allele, and adds three novel variants to the mutational spectrum. The findings support first-line *SLC4A11* testing in Saudi infants with congenital corneal clouding.

## 4. Methods

### 4.1. Study Design and Ethical Approval

This was a prospective study of patients with clinically diagnosed CHED recruited at the anterior segment clinic at King Khaled Eye Specialist Hospital and Research Center (KKESH & RC) in Riyadh, Saudi Arabia. The study followed the tenets of the Declaration of Helsinki and was approved by the Institutional Review Board (approval number RP 24177-P). Written informed consent was obtained from all participants, or from a parent or legal guardian for participants who were minors. The study was conducted from January 2025–May 2026.

### 4.2. Inclusion and Exclusion Criteria

Forty-seven Saudi patients from related Saudi families with clinically diagnosed CHED were prospectively recruited from the anterior segment clinics at King Khaled Eye Specialist Hospital (KKESH) for genetic screening. Inclusion criteria: Patients were eligible for inclusion if they had a clinical diagnosis of CHED based on characteristic ophthalmic findings, including bilateral, symmetrical corneal clouding with a ground-glass appearance, increased central corneal thickness, and disease onset at birth or during early infancy. Exclusion criteria: Patients with clinical features suggestive of primary congenital glaucoma or other congenital causes of corneal clouding were excluded. Specifically, CHED was distinguished by failure of corneal clearing despite adequate IOP control following topical or surgical management, a normal corneal diameter for age, and the absence of buphthalmos, Haab’s striae or Descemet membrane breaks. Examination under anesthesia was performed when required to establish the diagnosis [17].

### 4.3. Patients and Clinical Evaluation

Forty-seven Saudi patients from related Saudi families were enrolled. CHED was diagnosed on the basis of bilateral, symmetrical corneal clouding with a ground-glass appearance present at birth or in early infancy, increased central corneal thickness, and the absence of features indicating an alternative diagnosis. Each patient underwent a full ophthalmic examination, including slit-lamp biomicroscopy, intraocular pressure (IOP) measurement, central corneal pachymetry, MS-39 anterior segment optical coherence tomography [CSO Italia, Florence, Italy], specular microscopy [CEM-530 NIDEK Co., Ltd., Gamagori, Aichi, Japan], cycloplegic retinoscopy, and indirect ophthalmoscopy. Family history and consanguinity were recorded, and a pedigree was constructed for each family.

### 4.4. DNA Extraction

Peripheral venous blood was collected in EDTA tubes from affected individuals, available relatives, and controls. Genomic DNA was extracted from peripheral blood leukocytes using the QIAamp DNA Blood Mini Kit (Qiagen, Hilden, Germany; cat. no. 51106) according to the manufacturer’s protocol. Briefly, 200 µL of whole blood was lysed with proteinase K and buffer AL, then applied to a QIAamp spin column where DNA bound to the silica membrane. Bound DNA was washed with buffers AW1 and AW2 and eluted in 200 µL of buffer AE. DNA concentration and purity were assessed by spectrophotometry (NanoDrop, Thermo Fisher Scientific, Wilmington, Delaware, United States), and samples were stored at minus 20 °C until use.

### 4.5. Mutation Screening (Whole-Exome Sequencing)

All recruited patients went through whole-exome sequencing (WES). Peripheral venous blood was collected from the index case and available family members in EDTA tubes, and genomic DNA was isolated using a standard extraction protocol and quantified. The DNA was fragmented, and a sequencing library was prepared, after which the exons and adjacent splice site regions of the targeted genes were captured and enriched using (Roche KAPA HyperExome v2M, Mountain View, California, United States) and sequenced on the (MGI DNBSEQ, MGI DNBSEQ, MGI TECH, Shenzhen, Guangdong, China) platform to generate paired-end reads. Reads were aligned to the UCSC hg19 human reference genome using BWA v.0.7.17, and GATK v.4.1.9.0 was used for base quality score correction and for detection of single nucleotide variants (SNVs), insertions and deletions (indels), and genotypes, while Exome Depth was used to detect copy number variation at the exon level. Each sample was required to meet defined quality thresholds before analysis, with average sequencing depth above 210× and at least 98.7% of target bases covered at 20× or higher. Variants were annotated and filtered using (Golden Helix VarSeq), drawing on clinical information, population frequency databases, disease databases, and in silico prediction tools, with gene and variant nomenclature following the HUGO Gene Nomenclature Committee (HGNC) and the Human Genome Variation Society (HGVS), respectively. Candidate variants were assessed for pathogenicity using PolyPhen-2, SIFT, and MutationTaster, and their allele frequencies were checked against gnomAD, the Exome Sequencing Project (ESP), the 1000 Genomes Project, and a Saudi population database. Variants were then classified as pathogenic, likely pathogenic, of uncertain significance, likely benign, or benign according to the American College of Medical Genetics and Genomics and Association for Molecular Pathology (ACMG/AMP) 2015 guidelines and the recommendations of the ClinGen Sequence Variation Interpretation Working Group (https://clinicalgenome.org) [18]. All candidate variants identified by whole exome sequencing were confirmed by bidirectional Sanger sequencing, and parental and, where available, additional family member testing was performed to determine the phase of the variants and to establish the mode of inheritance, with all plausible inheritance patterns considered and each variant evaluated against the family history and clinical findings to assess pathogenicity and causality.

### 4.6. In Silico Analysis

The functional effect of identified variants was predicted with a panel of in silico tools, including SIFT, PolyPhen-2, PROVEAN, MutationTaster, and CADD for missense variants, and [a splice-site prediction tool, for example varSEAK] for variants affecting splicing. Effects on protein stability were assessed with [I-Mutant 2.0/DUET] and local flexibility with [DynaMut]. Evolutionary conservation of the affected residues was evaluated by multiple-sequence alignment of SLC4A11 orthologs using [Clustal Omega/ConSurf 1.2.4] [19].

### 4.7. Variant Classification

Variants were classified as pathogenic, likely pathogenic, uncertain significance, likely benign, or benign, per the guidelines established by the American College of Medical Genetics and Genomics and the Association for Molecular Pathology (ACMG/AMP) Richards et al. [18]. Each variant underwent assessment against established criteria for pathogenic and benign evidence, and the resulting codes were combined according to published scoring rules to reach a final classification. Population allele frequencies were obtained from gnomAD, with careful consideration of both global and relevant population subsets. Due to the recessive inheritance pattern of SLC4A11-related disease and the consanguineous background of our cohort, the PM2 criterion was applied for variants that were absent or present at very low frequencies in control populations. Additionally, BS1 and BA1 criteria were utilized to exclude alleles that were too common to potentially cause a fully penetrant recessive disorder. Since homozygosity is anticipated in consanguineous individuals, the mere observation of a variant in the homozygous state in an affected individual was not regarded as strong evidence alone; this was interpreted alongside phenotype and segregation data. For missense changes, computational predictions from multiple in silico tools were utilized as supporting evidence (PP3 or BP4), and considerations included nucleotide and amino acid conservation. Predicted splicing effects were assessed for variants located at or near canonical splice sites. Null variants, such as nonsense, canonical splice site, and frameshift changes, were assigned a PVS1 classification where loss of function is recognized as an established disease mechanism for SLC4A11. Previously reported variants were cross-checked against ClinVar and existing literature, and prior classifications were scrutinized rather than accepted without question. In cases where family material was available, co-segregation with disease was factored in. Variants classified as pathogenic or likely pathogenic were retained as candidate disease-causing alleles, while variants of uncertain significance were documented but interpreted with caution.

## 5. Conclusions

We characterized 47 consanguineous Saudi CHED patients by whole exome sequencing and found a homozygous *SLC4A11* variant in every one, with no compound heterozygotes. The 47 patients carried 20 unique variants, three of them novel, and the splice donor allele c.2018+1G>A alone accounted for 14 patients. The variants split into two mechanistic classes: missense substitutions predicted to misfold and be retained in the endoplasmic reticulum, and truncating or splice changes predicted to abolish the protein. Five of the nine missense variants were glycine-to-arginine substitutions clustered between residues 378 and 413 in the first transmembrane region, marking a structural hotspot. Because the pathogenic alleles concentrate in a few regions of the gene, a targeted Sanger protocol covering these hotspots would serve as an affordable first-line test in this population, with exome sequencing reserved for hotspot-negative cases.

## Figures and Tables

**Table 1:** Mutations detected in the *SLC4A11* gene in the CHED patients.

Patient ID	Sex	Age	*SLC4A11* Mutation	Mutation Type	Predicted Consequence of the Variant	HGMD	Pathogenicity
CHED-15	F	24	c.2018+1G>A	Splicing	Frameshift -Exon 16 skipping	CS095207	Likely Pathogenic
CHED-21	F	18	c.2018+1G>A	Splicing	Frameshift -Exon 16 skipping	CS095207	Likely Pathogenic
CHED-18	M	30	c.700G>T; p.V234F	Missense	Protein Misfolding	CM1911901	Likely Pathogenic
CHED-24	M	12	c.1201G>A; p.G401R	Missense	Damaged protein function	CM137450	Likely Pathogenic
CHED-16	M	29	c.1132G>C; p.G378R	Missense	Protein Misfolding	-	Pathogenic
CHED-09	M	12	c.1237G>A; p.G413R	Missense	Protein Misfolding	-	Likely Pathogenic
CHED-08	F	13	c.2188C>T; p.R730*	Nonsense	Premature protein termination	-	Pathogenic
CHED-03	F	17	**c.1799C>A; p.A600E**	**Missense**	**Protein Misfolding**	**-**	**VUS**
CHED-42	M	59	c.1189G>A; p.G397R	Missense	Protein Misfolding	CM1914336	Likely Pathogenic
CHED-44	M	41	c.1189G>A; p.G397R	Missense	Protein Misfolding	CM1914336	Likely Pathogenic
CHED-01	M	6	c.1180G>C; p.G394R	Missense	Disrupts TMD1	CM095208	Pathogenic
CHED-02	M	6	c.2018+1G>A; p.(?)	Splicing	Frameshift -Exon 16 skipping	CS095207	Likely Pathogenic
CHED-11	M	13	c.1180G>C; p.G394R	Missense	Disrupts TMD1	CM095208	Pathogenic
CHED-06	F	26	c.2140C>T; p.R714*	Nonsense	Premature stop-codon	CM095209	Pathogenic
CHED-07	M	23	c.1180G>C; p.G394R	Missense	Disrupts TMD1	CM095208	Pathogenic
CHED-13	M	11	c.1282+4C>T; p.(?)	Splicing	Significant impact on splicing	-	VUS
CHED-10	F	12	c.1282+1G>A; p.(?)	Splicing	Significant impact on splicing	-	Pathogenic
CHED-40	F	36	c.2018+1G>A; p.(?)	Splicing	Frameshift -Exon 16 skipping	CS095207	Likely Pathogenic
CHED-41	F	8	c.2018+1G>A; p.(?)	Splicing	Frameshift -Exon 16 skipping	CS095207	Likely Pathogenic
CHED-39	F	37	c.764C>T; p.T255M	Missense	Protein Misfolding	-	Pathogenic
CHED-38	F	28	c.425_432del; p.R142Qfs*4	Deletion	Premature stop-codon	CD072493	Pathogenic
CHED-37	M	16	c.700G>T; p.V234F	Missense	Protein Misfolding	CM1911901	Likely Pathogenic
CHED-36	F	16	c.2118C>A; p.Y706*	Nonsense	Premature stop-codon	-	Pathogenic
CHED-35	F	28	c.2285G>A; p.W762*	Nonsense	Premature stop-codon	-	Likely Pathogenic
CHED-34	M	22	c.2018+1G>A; p.(?)	Splicing	Frameshift -Exon 16 skipping	CS095207	Likely Pathogenic
CHED-33	F	10	c.2018+1G>A; p.(?)	Splicing	Frameshift -Exon 16 skipping	CS095207	Likely Pathogenic
CHED-31	M	11	c.700G>T; p.V234F	Missense	Protein Misfolding	CM1911901	Likely Pathogenic
CHED-30	M	4	c.2018+1G>A; p.(?)	Splicing	Frameshift -Exon 16 skipping	CS095207	Likely Pathogenic
CHED-27	F	15	c.1132G>C; p.G378R	Missense	Protein Misfolding	-	Pathogenic
CHED-28	F	11	c.1975G>A; p.E659K	Missense	Protein Misfolding	-	VUS
CHED-25	M	40	c.2018+1G>A; p.(?)	Splicing	Frameshift -Exon 16 skipping	CS095207	Likely Pathogenic
CHED-23	F	41	c.1132G>C; p.G378R	Missense	Protein Misfolding	-	Pathogenic
CHED-22	M	23	c.2018+1G>A; p.(?)	Splicing	Frameshift -Exon 16 skipping	CS095207	Likely Pathogenic
CHED-20	M	38	c.1132G>C; p.G378R	Missense	Protein Misfolding	-	Pathogenic
CHED-17	F	6	c.2018+1G>A; p.(?)	Splicing	Frameshift -Exon 16 skipping	CS095207	Likely Pathogenic
CHED-19	F	23	c.2018+1G>A; p.(?)	Splicing	Frameshift -Exon 16 skipping	CS095207	Likely Pathogenic
CHED-49	F	20	c.2147+1G>A; p.(?)	Splicing	Loss of canonical donor site	-	Likely Pathogenic
CHED-52	M	12	c.1180G>C; p.G394R	Missense	Disrupts TMD1	CM095208	Pathogenic
CHED-53	F	12	c.2623C>T; p.R875*	**Nonsense**	**Premature stop-codon**	**-**	**VUS**
CHED-54	F	12	c.1180G>C; p.G394R	Missense	Disrupts TMD1	CM095208	Pathogenic
CHED-32	F	8	c.700G>T; p.V234F	Missense	Protein Misfolding	CM1911901	Likely Pathogenic
CHED-48	M	28	c.2018+1G>A; p.(?)	Splicing	Frameshift -Exon 16 skipping	CS095207	Likely Pathogenic
CHED-43	F	9	c.670dup; p.W224Lfs*7	**Duplication**	**Frameshift -Loss of function**	**-**	**Likely Pathogenic**
CHED-46	F	37	c.1132G>C; p.G378R	Missense	Protein Misfolding	-	Pathogenic
CHED-47	M	20	c.2140C>T; p.R714*	Nonsense	Premature stop-codon	CM095209	Pathogenic
CHED-12	F	12	c.764C>T; p.T255M	Missense	Protein Misfolding	-	Pathogenic
CHED-14	M	12	c.2018+1G>A; p.(?)	Splicing	Frameshift -Exon 16 skipping	CS095207	Likely Pathogenic

The *SLC4A11* gene (Transcript NM_032034.4) was linked to all variations. Variants found in CHED patients (*n* = 47) are shown in the above table. Every variation found was homozygous. Bold text indicates novel variants.

**Table 2:** Mechanistic classification of the 20 unique *SLC4A11* (NM_032034.4) variants detected in 47 CHED patients.

Variant	Protein	Type	Residue and Domain	Predicted Effect on Protein	Patients (n)	Pathogenicity
**Class** **1. Folding and trafficking failure (9 variants, 22 patients)**						
c.700G>T	p.V234F	Missense	234, cytoplasmic domain	Misfolding of the cytoplasmic domain, predicted ER retention	4	Likely pathogenic
c.764C>T	p.T255M	Missense	255, cytoplasmic domain	Misfolding of the cytoplasmic domain, predicted ER retention	2	Pathogenic
c.1132G>C	p.G378R	Missense	378, start of the membrane domain, TM1 boundary	Charged residue introduced at the start of TM1, predicted ER retention	5	Pathogenic
c.1180G>C	p.G394R	Missense	394, membrane domain, TM1 region	Charged residue introduced in the TM1 region, predicted ER retention	5	Pathogenic
c.1189G>A	p.G397R	Missense	397, membrane domain, TM1 region	Charged residue introduced in the TM1 region, predicted ER retention	2	Likely pathogenic
c.1201G>A	p.G401R	Missense	401, membrane domain, TM1 region	Charged residue introduced in the TM1 region, predicted ER retention	1	Likely pathogenic
c.1237G>A	p.G413R	Missense	413, membrane domain, TM1 region	Charged residue introduced in the TM1 region, predicted ER retention	1	Likely pathogenic
c.1799C>A	p.A600E	Missense	600, membrane domain	Charged residue introduced in the membrane domain, predicted misfolding and ER retention	1	VUS
c.1975G>A	p.E659K	Missense	659, membrane domain	Charge reversal in the membrane domain, predicted misfolding and ER retention	1	VUS
**Class 2. Absent or truncated protein (11 variants, 25 patients)**						
c.425_432del	p.R142Qfs*4	Frameshift deletion	142, cytoplasmic domain	Premature termination codon, predicted nonsense-mediated decay	1	Pathogenic
c.670dup	p.W224Lfs*7	Frameshift duplication	224, cytoplasmic domain	Premature termination codon, predicted nonsense-mediated decay	1	Likely pathogenic
c.2118C>A	p.Y706*	Nonsense	706, membrane domain	Premature termination codon, loss of the C-terminal transmembrane helices, predicted decay	1	Pathogenic
c.2140C>T	p.R714*	Nonsense	714, membrane domain	Premature termination codon, loss of the C-terminal transmembrane helices, predicted decay	2	Pathogenic
c.2188C>T	p.R730*	Nonsense	730, membrane domain	Premature termination codon, loss of the C-terminal transmembrane helices, predicted decay	1	Pathogenic
c.2285G>A	p.W762*	Nonsense	762, membrane domain	Premature termination codon, loss of the C-terminal transmembrane helices, predicted decay	1	Likely pathogenic
c.2623C>T	p.R875*	Nonsense	875, membrane domain, C terminus	Premature termination near the C terminus, predicted to escape nonsense-mediated decay, loss of the final 17 residues	1	VUS
c.1282+1G>A	p.(?)	Splicing	Canonical donor, +1	Loss of the canonical splice donor, predicted aberrant splicing and frameshift	1	Pathogenic
c.1282+4C>T	p.(?)	Splicing	Donor region, +4	Predicted weakening of the donor site, consequence unconfirmed	1	VUS
c.2018+1G>A	p.(?)	Splicing	Canonical donor, +1	Loss of the canonical splice donor, reported exon 16 skipping and frameshift, unconfirmed in this cohort	14	Likely pathogenic
c.2147+1G>A	p.(?)	Splicing	Canonical donor, +1	Loss of the canonical splice donor, predicted aberrant splicing and frameshift	1	Likely pathogenic

Variants are numbered against *SLC4A11* transcript NM_032034.4, protein NP_114420.2 (891 residues). Domain assignment follows the published topology, in which residues 1 to 374 form the N-terminal cytoplasmic domain and residues 375 to 891 form the membrane domain.

## Data Availability

No new data were created or analyzed in this study. Data sharing is not applicable to this article.

## Abbreviations

The following abbreviations are used in this manuscript:

ACMG	American College of Medical Genetics and Genomics
AMP	Association for Molecular Pathology
BCVA	Best corrected visual acuity
BTR1	Bicarbonate transporter related protein 1
BWA	Burrows-Wheeler Aligner
CADD	Combined Annotation Dependent Depletion
CHED	Congenital hereditary endothelial dystrophy
DNA	Deoxyribonucleic acid
DSAEK	Descemet stripping automated endothelial keratoplasty
EDTA	Ethylenediaminetetraacetic acid
ER	Endoplasmic reticulum
ESP	Exome Sequencing Project
FECD	Fuchs endothelial corneal dystrophy
GATK	Genome Analysis Toolkit
gnomAD	Genome Aggregation Database
HGMD	Human Gene Mutation Database
HGVS	Human Genome Variation Society
IC3D	International Classification of Corneal Dystrophies
indel	Insertion or deletion
IOP	Intraocular pressure
KKESH	King Khaled Eye Specialist Hospital
MRN	Medical record number
OCT	Optical coherence tomography
OD	Oculus dexter, right eye
OS	Oculus sinister, left eye
OU	Oculus uterque, both eyes
PCG	Primary congenital glaucoma
PPCD	Posterior polymorphous corneal dystrophy
RNA	Ribonucleic acid
SLC4A11	Solute carrier family 4 member 11
SNV	Single nucleotide variant
**TM1**	Transmembrane region 1
**VUS**	Variant of uncertain significance
